# A framework for integrating wastewater-based epidemiology and public health

**DOI:** 10.3389/fpubh.2024.1418681

**Published:** 2024-07-24

**Authors:** Hanna Brosky, Sarah M. Prasek, Gabriel K. Innes, Ian L. Pepper, Jasmine Miranda, Paul E. Brierley, Stephanie L. Slinski, Lois Polashenski, Walter Q. Betancourt, Katie Gronbach, Diana Gomez, Reshma Neupane, Jasmine Johnson, Joli Weiss, Hayley D. Yaglom, David M. Engelthaler, Crystal M. Hepp, Katherine Crank, Daniel Gerrity, Jill R. Stewart, Bradley W. Schmitz

**Affiliations:** ^1^Yuma Center of Excellence for Desert Agriculture (YCEDA), University of Arizona, Tucson, AZ, United States; ^2^Environmental Sciences and Engineering, University of North Carolina, Chapel Hill, NC, United States; ^3^Water and Energy Sustainable Technology (WEST) Center, University of Arizona, Tucson, AZ, United States; ^4^Yuma County Public Health Services District, Yuma, AZ, United States; ^5^Arizona Department of Health Services, Office of Infectious Disease Services, Phoenix, AZ, United States; ^6^Translational Genomics Research Institute, Pathogen and Microbiome Institute, Flagstaff, AZ, United States; ^7^Applied Research and Development Center, Southern Nevada Water Authority, Las Vegas, NV, United States

**Keywords:** wastewater-based epidemiology, public health, response actions, framework, environmental surveillance

## Abstract

Wastewater-based epidemiology (WBE) is an environmental approach to monitor community health through the analysis of sewage. The COVID-19 pandemic catalyzed scientists and public health professionals to revisit WBE as a tool to optimize resource allocation to mitigate disease spread and prevent outbreaks. Some studies have highlighted the value of WBE programs that coordinate with public health professionals; however, the details necessary for implementation are not well-characterized. To respond to this knowledge gap, this article documents the framework of a successful WBE program in Arizona, titled Wastewater Analysis for Tactical Epidemiological Response Systems (WATERS), detailing the developed structure and methods of communication that enabled public health preparedness and response actions. This communication illustrates how program operations were employed to reduce outbreak severity. The structure outlined here is customizable and may guide other programs in the implementation of WBE as a public health tool.

## Graphical abstract

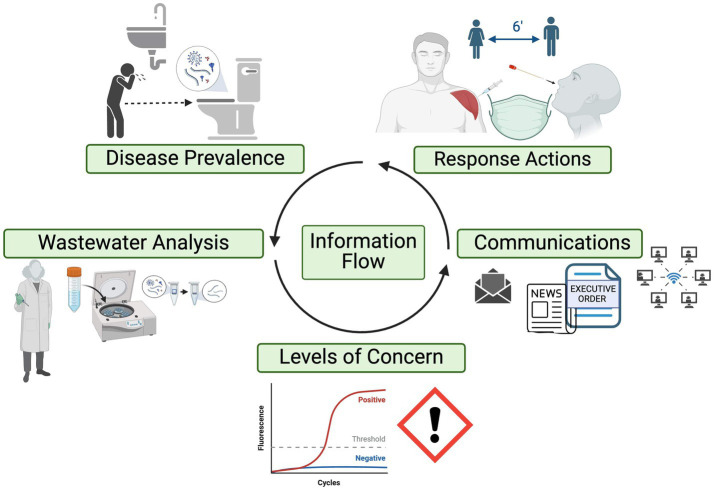

## 1 Introduction

Wastewater-based epidemiology (WBE) is a public health surveillance tool that uses measured concentrations of constituents within sewage to assess a community’s underlying health status (1–4). After initiation during the Global Polio Eradication Initiative, WBE regained popularity during the SARS-CoV-2 pandemic as an early-warning alert system for outbreaks and to assist in efficient resource allocation and hotspot identification (5). Much of the relevant literature has focused on technical methods to process wastewater samples (6, 7); measure disease biomarkers (8, 9); correlate data with health conditions in the sampled population (10, 11); and interpret results for stakeholders (12, 13). However, few studies have described the complex coordination structure needed to build a successful WBE program capable of guiding public health response and decision-making to mitigate disease spread and minimize outbreaks (14).

During the COVID-19 pandemic, the Centers for Disease Control and Prevention (CDC) established the National Wastewater Surveillance System (NWSS) to monitor disease trends across the United States using wastewater data (15). The crucial steps for ensuring a sustainable NWSS program were highlighted by the National Academies’ (NA) report entitled *Wastewater-based Disease Surveillance For Public Health Action* (16). This report posited the importance of creating a partnership among local, regional, and federal jurisdictions, and underscored the value of multidisciplinary and transdisciplinary collaboration (e.g., municipal utilities, public health agencies, academics, and industry). This report details the complexities of WBE and essential components of establishing a WBE program, without describing how to integrate public health action based upon wastewater findings.

This communication aims to describe the structure and operation of a municipal WBE program in Arizona that has successfully informed decision-makers and guided actions to minimize and avert disease outbreaks. With close to a decade of experience in WBE (17), our team was among the pioneers in detecting SARS-CoV-2 in wastewater during the COVID-19 pandemic (18). This expertise enabled the development of a WBE framework titled WATERS (Wastewater Analysis for Tactical Epidemiological Response Systems). The structure outlined here serves as a practical and adaptable resource to assist those developing similar public health programs.

## 2 Program structure—municipal WBE

To establish a municipal WBE program in Yuma County, Arizona, the University of Arizona (UArizona) quickly sought the approval and investment of local utility representatives, public health leadership, and other community entities (Table 1). In this case, the lead organization was the UArizona; however, that role could be assumed by any stakeholder. Memoranda of Understanding (MOUs) (see Supplementary material) were drafted among the Yuma County Public Health Services District (YCPHSD) and WBE program partners to define each organization’s commitments. Two primary groups were organized to implement the WBE program: the Local Surveillance and Response Teams (LSRTs) (see section 2.1) and a Steering Committee (see section 2.2). A communication network, including methods for regularly sharing data with partners and feedback for program adjustment was also developed.

**Table 1:** Roles and responsibilities of the Yuma County, Arizona WBE team.

Stakeholder	Responsibility
Wastewater utilities	Collect and deliver wastewater samples Identify potential wastewater sampling locations Participate in LSRT and Steering Committee
Laboratory (academic, industry, etc.)	Conduct wastewater testing Interpret and communicate results across stakeholders Participate in LSRT and Steering Committee
Municipalities	Inform stakeholders of key events Supply public outreach Provide decision-making for public health actions Participate in LSRT and Steering Committee
Public Health Officials (local)	Inform stakeholders of clinical data Provide public facing dashboards Provide resources and enact response actions (testing, vaccinations) Participate in LSRT and Steering Committee
Public Health Officials (state and federal)	Generate public facing dashboard (wastewater and clinical data) Provide resources for action Participate in Steering Committee
Schools/Universities	Inform stakeholders about events Provide student health updates Participate in Steering Committee
Medical Centers	Maintain clinical responsibilities Provide population health updates Participate in Steering Committee
Local businesses/industry	Inform stakeholders about events Identify building-level testing sites (optional)
Lead Organization	Lead program coordination Host Steering Committee meetings Develop public health models
*Any organization can be the lead*	

This team serves as a model for the Wastewater Analysis for Tactical Epidemiological Response Systems (WATER) framework.

### 2.1 Local surveillance and response teams

**Figure 1:**
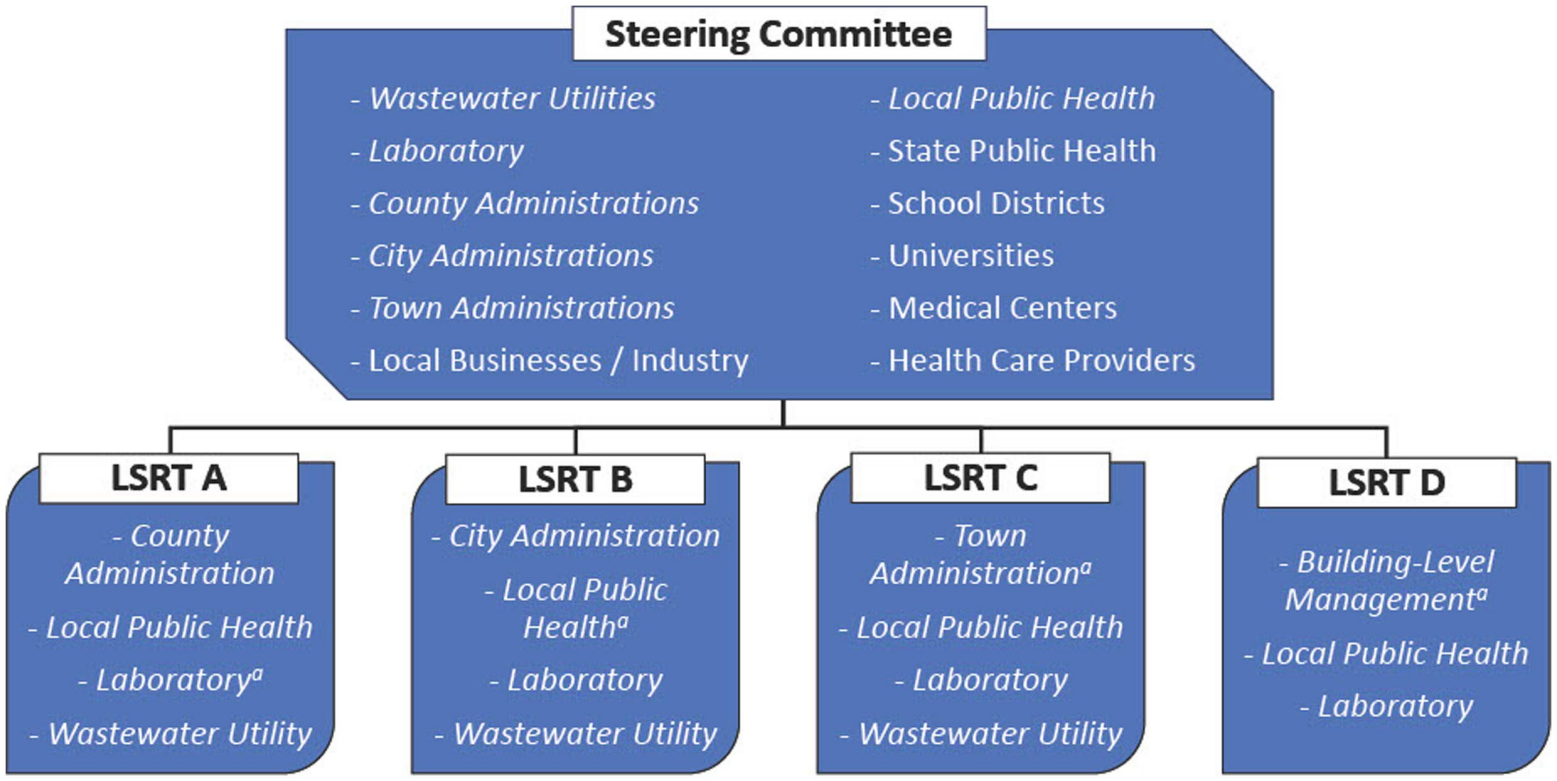
Example of a Local Surveillance and Response Teams (LSRT) and Steering Committee members within the WATERS framework. *Italicized* text signifies both in Steering Committee and LSRTs. ^a^The lead or “champion” denoted in each LSRT.

The LSRTs were established to facilitate rapid exchange of information, allowing for targeted decision making related to WBE testing and response. These groups typically consisted of a “champion” and three to four members from key organizations within specific communities (Figure 1). In coordination, LSRT members worked cohesively to identify sampling locations, and laboratory staff processed wastewater samples within a 24 h period. LSRTs received immediate result interpretations from the laboratory for their respective municipality, and met to determine if response was necessary, whereupon, members decided on resource allocation and how to execute an effective response. LSRTs only made decisions regarding their own community and utilized readily available resources (e.g., mask mandate). If additional resources were needed, LSRTs coordinated with the Steering Committee for support (see Section 3.1).

### 2.2 WBE steering committee

The Steering Committee was organized to coordinate the WBE program across jurisdictions. This group brought together wastewater result interpretations and disease status information from the multiple LSRTs; discussed regional trends and events; managed resource sharing among communities; and developed public outreach communications which LSRTs could subsequently distribute. If more resources were required, the Steering Committee would make a request to state or federal agencies on behalf of stakeholders and communities. This committee consisted of numerous key decision-makers (i.e., 36 members in Yuma County), including representatives from the LSRTs and various stakeholder groups (Figure 1). The Steering Committee engaged in weekly communications to ensure a proactive approach for information sharing to enable LSRTs to execute public health actions when necessary.

### 2.3 Communications

To facilitate data interpretation and determine appropriate response actions, a metric called “Levels of Concern” (LOC), was developed (Table 2). These levels were designed upon a baseline understanding of the relationship between SARS-CoV-2 RNA concentrations in wastewater and reported number of infections (symptomatic and asymptomatic) within a controlled population (19). Levels 0 and 1 indicated low disease incidence and prompted continued wastewater monitoring. Levels 2 and 3 indicated potential elevated disease incidence and were paired with considerations for public health preparedness and response actions.

**Table 2:** Levels of concern (LOC) developed to facilitate communication and prompt public health actions based on wastewater.

Laboratory results (Two or more consecutive samples must meet criteria)		Level of concern	Interpretation	Public health considerations	Communication threshold
RT-qPCR	Digital PCR				
Mean [Conc.]^a^: non-detect C_q_ value: all wells >37; <LoD Short-term alert^b^: negative Long-term alert^c^: negative	Both undiluted AND 1:10 diluted sample: <3 positive partitions	**0**	No evidence of infection	No response indicated	—
Mean [Conc.]: <104 Cq value: 1 well <37 Short-term alert OR long-term alert: positive	Undiluted sample OR 1:10 diluted sample: 3–9 positive partitions	**1**	Low disease prevalence	Continue routine wastewater monitoring Passive clinical monitoring from healthcare and syndromic data	—
Mean [Conc.]: 10^4^–10^5.9^ C_q_ value: two wells <37 Short-term alert AND/OR long-term alert: positive	Undiluted sample: 10–99 positive partitions, OR 1:10 diluted sample: 10–99 positive partitions, OR Undiluted sample AND 1:10 diluted sample: 3–9 positive partitions	**2**	Moderate disease prevalence	Employ LOC 1 actions Alert notifications to public Contact public health partners Alert local healthcare facilities (including acute care and long-term care settings) Infection control education (e.g., masking and social distancing)	Jurisdictional Public Health Partners, Consider additional stakeholders
Mean [Conc.]: >10^6^ C_q_ value: three wells <37 Short-term alert AND long-term alert: positive	Both undiluted AND 1:10 diluted sample: >100 positive partitions	**3**	High disease prevalence	Employ LOC 2 actions Ensure availability of healthcare resources (e.g., human resources, PPE, ventilators) Remote work for non-essential personnel Masking in indoor venues, including schools Targeted wastewater testing at the building level	Jurisdictional Public Health Partners, Steering Committee members, and consider additional stakeholders

^a^Mean [Conc.]: Mean of SARS-CoV-2 N1 RNA concentration from sampled wastewater averaged across three PCR wells.

^b^Short-term alert is defined as the concentration of SARS-CoV-2 N1 RNA is ≥ 130% mean concentration of RNA analyzed from the previous 5 sampling days.

^c^Long-term alert is defined as the concentration of SARS-CoV-2 N1 RNA is ≥ 130% mean concentration of RNA in analyzed from the previous 30 days.Bolded numbers represent interpretations of the laboratory results in a leikert-esque format. The numbers should not be confused as a measurement in itself.

Local Surveillance and Response Teams received standard email communications which included data interpretations from sampling events, the LOC chart, and a liability disclaimer (see Supplementary material). Based on the observed SARS-CoV-2 RNA concentrations and the established LOCs, LSRT members communicated to reach consensus and, when needed, implemented appropriate response actions for their community (Table 2). A summary of data interpretations and recent events for each LSRT were emailed to the broader Steering Committee on a weekly basis, and members of this group met virtually, depending on the state of emergency. If LOCs reached level 2 and/or 3 across all jurisdictions, Steering Committee members received an immediate alert in addition to the weekly communication email. When necessary, LSRTs and/or the Steering Committee met to discuss health alert notifications for relevant populations in addition to neighboring jurisdictions that might experience similar infection rates due to potential disease spread.

## 3 Integrating WBE and public health

### 3.1 Municipal public health actions

Multi-directional information flow ensured that stakeholders within the LSRTs and the Steering Committee were informed of changing priorities and could respond promptly to developing conditions (Figure 1). Applied to several municipalities, this WBE framework encouraged frequent conversations outside of formal settings between local public health officials and jurisdictional leadership.

For example, after receiving notification of increasing LOC (Table 2), one city administrator requested that wastewater be collected at additional sampling sites to provide more granular level community information (20). This expansion of wastewater sampling resulted in the identification of neighborhoods that were likely experiencing an increased incidence of COVID-19 infections. City officials were then able to concentrate efforts to improve health conditions in those neighborhoods, enhancing vaccination efforts, testing measures, and public notifications, to optimize resource allocation (see Supplementary Figures S1–S6).

In other instances, this collaboration’s efforts led to the adoption of mask mandates which cited WBE results and increased clinical cases as the rationale for elevated caution (21). Also, in partnership with the Translational Genomics Research Institute (TGen), the laboratory detected COVID-19 variants before local cases were reported (22). Early identification helped to inform public health entities of novel variants and a potential increase in infections. As an example, the identification of Omicron mobilized LSRTs and the Steering Committee to enact public health interventions for disease containment (e.g., school notification, increased workplace PPE, road sign public health messaging, media) (23).

### 3.2 WBE program structure adaptability

The WBE program structure was adaptable across diverse settings. Overall, organizing principles guided program establishment and configuration, while the stakeholders and system of communication were adjustable depending on the needs and scale of each community (Table 3).

**Table 3:** The Arizona WBE framework is scalable, with applications already demonstrated across building-level, university, and municipal scaled in Arizona.

Size	Municipality	University	Building-level
LSRT “Champion”	Laboratory	Affiliated Faculty	Building-level staff
Decision Makers	Jurisdictional Representatives, Local Public Health	University Leadership	Building-level leadership
Communication Method	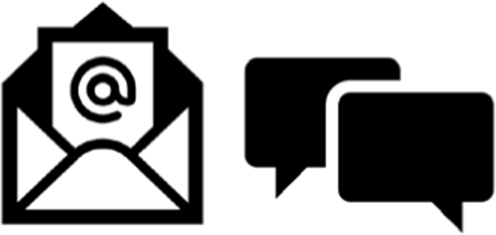	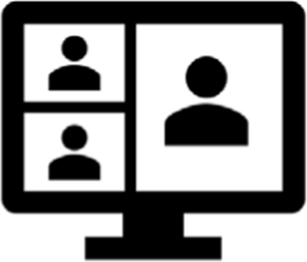	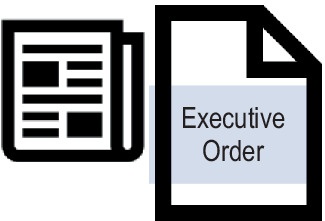
	Email and ad-hoc conversations	Virtual meeting	Public communications
Interventions	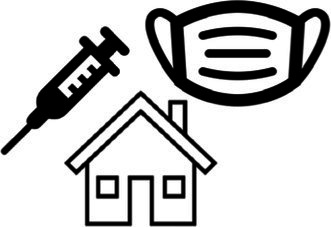	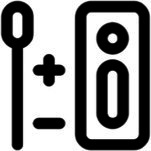	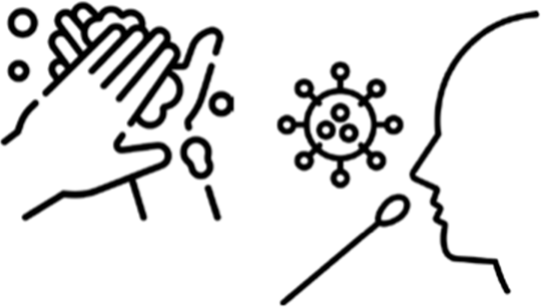
	Vaccinations	Clinical testing kits	Personal hygiene

For instance, UArizona established a WBE program to reduce the pandemic’s impact on the university’s campus community. Similar to the municipality WBE program, the university’s program coupled analytical results from wastewater samples with a system of communication and toolbox of potential interventions. Similarly, the collaborative WBE structure was adapted to ensure worker health and safety in the agriculture and food sector, as described for a date fruit packing facility (24). In this example, the size of the facility’s WBE program was small (i.e., building specific), allowing a streamlined LSRT and Steering Committee which amounted to a single decision-making body. Employing wastewater surveillance in participating facilities produced rapid detection of asymptomatic individuals, and effective intervention deployment. Finally, the WBE structure was adapted to monitor wastewater of incarcerated individuals at a detention center to prevent disease within the institution (data not publicly available). These applications demonstrate scalability of the developed framework.

Beyond the ability to adapt to many population sizes and demographics (Table 3), the WBE framework is not bounded by geography or disease target. Additionally, communication methods to report wastewater results and subsequent interventions are not limited to specific scales (municipality, university, and building) (Table 3). The structure developed in Arizona has informed the implementation of successful local programs in three additional states [Louisiana (18), Florida (25), and Virginia (26)]. Additionally, the LOCs can be adapted to fit the wastewater target of interest. For instance, wastewater monitoring of *Candida auris* in Las Vegas, Nevada (27) has provided a baseline for developing LOCs specific to this opportunistic fungal pathogen (Supplementary Table S1).

## 4 Discussion

The described WBE framework in Arizona served as a practical tool that helped limit disease transmission. WBE cannot differentiate between asymptomatic and symptomatic infections because both result in viral shedding; therefore, a key strength is that wastewater analysis can provide insight into the overall health of a population (28). The organizational structure delineations, such as the Steering Committee and the LSRTs, facilitated rapid and evidence-driven decision making and the efficient delivery of information to stakeholders, policy makers, and others. The LSRTs comprised small groups to guarantee expeditious responses and limit the number of decision-makers. These same members were included in the larger Steering Committee to provide multiple points of interaction and knowledge sharing. The operations of these communication networks were flexible: communication frequency; sampling plans; and interventions evolved throughout the pandemic. Through this evolution, program participants were able to tailor their site-specific programs to address their community’s needs. This encouraged overall engagement in, and commitment to, the WBE project.

Ensuring stakeholder investment was essential to the success of Arizona’s WBE programs. The framework provided dependable information, communication, and response actions across all participants. This generated stakeholder buy-in and prevented a loss of interest and/or “burn-out” as the pandemic evolved. Additionally, stakeholders frequently utilized the program’s network to engage in impromptu discussions, which significantly enhanced overall community response.

While implementing the WBE framework, communities faced challenges. In particular, given that Yuma County was a COVID-19 hotspot (29) and the pandemic spanned multiple years, ongoing messaging and interventions resulted in community fatigue. Subsequently, LSRTs and the Steering Committee rapidly assessed and modified response actions to meet community needs and tolerances. For example, early communications faced community scrutiny regarding the inclusion of the word “wastewater.” People presumed infectious SARS-CoV-2 virus was present in the drinking water system. Communications were amended for clarity and instead used “sewage” to prevent confusion. This process of adaptation had the potential, at times, to require more coordination; however, the outcome was typically greater consensus and community support. Communications such as social media posts, television/radio broadcasts, street signs, and press releases (Supplementary Figures S1–S6) promoted behavior changes such as mask wearing, social distancing, clinical testing, vaccinations, and other appropriate public health interventions.

Ethical considerations are paramount when constructing and considering consequences of wastewater surveillance programs. Researchers have weighed the ethics involved in wastewater testing (30); however, guidelines are not standardized or adopted across wastewater surveillance programs. Individuals involved in wastewater programs should consider the purpose and methodology behind wastewater data collection, the utilization and communication of this information to the contributing community members, and the equitable distribution of any resulting interventions (31). Ethics were incorporated at various scales within the WATERS framework. At the municipal level, LSRTs included *ad hoc* discussions regarding ethics, and actions guided by wastewater results were with public health protection at the forefront. At the building level, wastewater data was not capable of identifying specific individuals or groups. Participation with clinical testing and vaccinations was strictly voluntary, and the university’s Institutional Review Board (IRB) verified that all data was de-identified and complied with the Human Subjects Protection Program (13).

## 5 Conclusion

Although WBE has gained traction since the COVID-19 pandemic, to the best of our knowledge, this is the first paper to detail explicitly an operational system for integrating WBE into public health action. The success of this WBE framework can be attributed largely to effective collaboration and communication within LSRTs and Steering Committees to inform prompt deployment of public health interventions. Program adaptability is a crucial element necessary for municipalities initiating an effective WBE program. This WATERS framework serves as a blueprint for communities planning to integrate wastewater data into public health decision-making.

## Data availability statement

The raw data supporting the conclusions of this article will be made available by the authors, without undue reservation.

## Author contributions

HB: Formal analysis, Visualization, Writing – original draft, Writing – review & editing. SP: Formal analysis, Writing – original draft, Writing – review & editing. GI: Conceptualization, Writing – review & editing. IP: Conceptualization, Writing – review & editing. JM: Data curation, Formal analysis, Writing – review & editing. PB: Conceptualization, Funding acquisition, Supervision, Writing – review & editing. SS: Funding acquisition, Project administration, Writing – review & editing. LP: Formal analysis, Methodology, Writing – review & editing. WB: Methodology, Writing – review & editing. KG: Formal analysis, Writing – review & editing. DGo: Resources, Writing – review & editing. RN: Resources, Writing – review & editing. JJ: Resources, Writing – review & editing. JW: Resources, Writing – review & editing. HY: Formal analysis, Writing – review & editing. DE: Formal analysis, Writing – review & editing. CH: Formal analysis, Writing – review & editing. KC: Methodology, Writing – review & editing. DGe: Methodology, Writing – review & editing. JS: Writing – review & editing. BS: Conceptualization, Funding acquisition, Investigation, Methodology, Project administration, Supervision, Writing – original draft, Writing – review & editing.

## References

[1] World Health Organization (2023). Environmental surveillance for SARS-CoV-2 to complement other public health surveillance.

[2] Fox SJ Lachmann M Tec M Pasco R Woody S du Z. Real-time pandemic surveillance using hospital admissions and mobility data. Proc Natl Acad Sci. (2022) 119:e2111870119. doi: 10.1073/pnas.2111870119, PMID: 35105729 PMC8851544

[3] Bibby K Bivins A Wu Z North D. Making waves: plausible lead time for wastewater based epidemiology as an early warning system for COVID-19. Water Res. (2021) 202:117438. doi: 10.1016/j.watres.2021.117438, PMID: 34333296 PMC8274973

[4] Gonzalez R Curtis K Bivins A Bibby K Weir MH Yetka K. COVID-19 surveillance in Southeastern Virginia using wastewater-based epidemiology. Water Res. (2020) 186:116296. doi: 10.1016/j.watres.2020.116296, PMID: 32841929 PMC7424388

[5] O'Reilly KM Allen DJ Fine P Asghar H. The challenges of informative wastewater sampling for SARS-CoV-2 must be met: lessons from polio eradication. Lancet Microb. (2020) 1:e189–90. doi: 10.1016/S2666-5247(20)30100-2, PMID: 32838348 PMC7386849

[6] Augusto MR Claro ICM Siqueira AK Sousa GS Caldereiro CR Duran AFA. Sampling strategies for wastewater surveillance: evaluating the variability of SARS-COV-2 RNA concentration in composite and grab samples. J Environ Chem Eng. (2022) 10:107478. doi: 10.1016/j.jece.2022.107478, PMID: 35251931 PMC8882035

[7] Ahmed W Bivins A Bertsch PM Bibby K Gyawali P Sherchan SP. Intraday variability of indicator and pathogenic viruses in 1-h and 24-h composite wastewater samples: implications for wastewater-based epidemiology. Environ Res. (2021) 193:110531. doi: 10.1016/j.envres.2020.110531, PMID: 33249042 PMC8267967

[8] Ando H Murakami M Ahmed W Iwamoto R Okabe S Kitajima M. Wastewater-based prediction of COVID-19 cases using a highly sensitive SARS-CoV-2 RNA detection method combined with mathematical modeling. Environ Int. (2023) 173:107743. doi: 10.1016/j.envint.2023.107743, PMID: 36867995 PMC9824953

[9] Rainey AL Liang S Bisesi JH Jr Sabo-Attwood T Maurelli AT. A multistate assessment of population normalization factors for wastewater-based epidemiology of COVID-19. PLoS One. (2023) 18:e0284370. doi: 10.1371/journal.pone.0284370, PMID: 37043469 PMC10096268

[10] Bertels X Demeyer P Van Den Bogaert S Boogaertsc T van Nuijsc ALN Delputteb P. Factors influencing SARS-CoV-2 RNA concentrations in wastewater up to the sampling stage: a systematic review. Sci Total Environ. (2022) 820:153290. doi: 10.1016/j.scitotenv.2022.153290, PMID: 35066048 PMC8772136

[11] Olesen SW Imakaev M Duvallet C. Making waves: defining the lead time of wastewater-based epidemiology for COVID-19. Water Res. (2021) 202:117433. doi: 10.1016/j.watres.2021.117433, PMID: 34304074 PMC8282235

[12] O’Keeffe J. Wastewater-based epidemiology: current uses and future opportunities as a public health surveillance tool. Environ Health Rev. (2021) 64:44–52. doi: 10.5864/d2021-015

[13] Parkins MD Lee BE Acosta N Bautista M Hubert CRJ Hrudey SE. Wastewater-based surveillance as a tool for public health action: SARS-CoV-2 and beyond. Clin Microbiol Rev. (2024) 37:e00103–22. doi: 10.1128/cmr.00103-22 38095438 PMC10938902

[14] McClary-Gutierrez JS Mattioli MC Marcenac P Silverman AI Boehm AB Bibby K. SARS-CoV-2 wastewater surveillance for public health action. Emerg Infect Dis. (2021) 27:1–8. doi: 10.3201/eid2709.210753 PMC8386792 34424162

[15] Center for Disease Control and Prevention (2020). National Wastewater Surveillance System (NWSS). Available at: https://www.cdc.gov/nwss/wastewater-surveillance.html#:~:text=In%20response%20to%20the%20COVID,samples%20collected%20across%20the%20country (Accessed March 14, 2023).

[16] National Academies of Sciences, Engineering, and Medicine. Wastewater-Based Disease Surveillance for Public Health Action. Washington, DC: The National Academies Press (2023). 37184191

[17] Schmitz BW Kitajima M Campillo ME Gerba CP Pepper IL. Virus reduction during advanced Bardenpho and conventional wastewater treatment processes. Environ Sci Technol. (2016) 50:9524–32. doi: 10.1021/acs.est.6b01384, PMID: 27447291

[18] Sherchan SP Shahin S Ward LM Tandukar S Aw TG Schmitz B. First detection of SARS-CoV-2 RNA in wastewater in North America: a study in Louisiana, USA. Sci Total Environ. (2020) 743:140621. doi: 10.1016/j.scitotenv.2020.140621, PMID: 32758821 PMC7833249

[19] Betancourt WQ Schmitz BW Innes GK Prasek SM Pogreba Brown KM Stark ER. COVID-19 containment on a college campus via wastewater-based epidemiology, targeted clinical testing and an intervention. Sci Total Environ. (2021) 779:146408. doi: 10.1016/j.scitotenv.2021.146408, PMID: 33743467 PMC7954642

[20] Albright A. (2021). Foothills wastewater testing shows rise in COVID-19 cases. Available at: https://kyma.com/news/2021/02/22/foothills-wastewater-testing-shows-rise-in-covid-19-cases/

[21] Hettinger A. (2021). San Luis mayor Gerardo Sánchez personally justifies reinstating mask mandate. Kyma. Available at: kyma.com

[22] Prasek SM Pepper IL Innes GK Slinski S Betancourt WQ Foster AR. Variant-specific SARS-CoV-2 shedding rates in wastewater. Sci Total Environ. (2023) 857:159165. doi: 10.1016/j.scitotenv.2022.159165, PMID: 36195153 PMC9527179

[23] Hettinger A. (2021). Home grown: Ag technology used for wastewater COVID detection. Available at: https://kyma.com/news/home-grown/2021/02/22/home-grown-ag-technology-used-for-wastewater-covid-detection/

[24] Innes GK Schmitz BW Brierley PE Guzman J Prasek SM Ruedas M. Wastewater-based epidemiology mitigates COVID-19 outbreaks at a food processing facility near the Mexico-U.S. border—November 2020–march 2022. Viruses. (2022) 14:2684. doi: 10.3390/v14122684, PMID: 36560688 PMC9786163

[25] Prasek SM Pepper IL Innes GK Slinski S Ruedas M Sanchez A. Population level SARS-CoV-2 fecal shedding rates determined via wastewater-based epidemiology. Sci Total Environ. (2022) 838:156535. doi: 10.1016/j.scitotenv.2022.156535, PMID: 35688254 PMC9172256

[26] Sherchan SP Shahin S Patel J Ward LM Tandukar S Uprety S. Occurrence of SARS-CoV-2 RNA in six municipal wastewater treatment plants at the early stage of COVID-19 pandemic in the United States. Pathogens. (2021) 10:798. doi: 10.3390/pathogens10070798, PMID: 34201687 PMC8308538

[27] Barber C Crank K Papp K Innes GK Schmitz BW Chavez J. Community-scale wastewater surveillance of Candida auris during an ongoing outbreak in southern Nevada. Environ Sci Technol. (2023) 57:1755–63. doi: 10.1021/acs.est.2c07763, PMID: 36656763 PMC9893721

[28] Laicans J Dejus B Dejus S Juhna T. Precision and accuracy limits of wastewater-based epidemiology—lessons learned from SARS-CoV-2: a scoping review. WaterSA. (2024) 16:1220. doi: 10.3390/w16091220

[29] AZCentral (2020). Yuma and Santa Cruz counties are COVID-19 hot spots in Arizona. Arizona Republic: azcentral. Available at: azcentral.com

[30] Hrudey SE Silva DS Shelley J Pons W Isaac-Renton J Chik AHS. Ethics guidance for environmental scientists engaged in surveillance of wastewater for SARS-CoV-2. Environ Sci Technol. (2021) 55:8484–91. doi: 10.1021/acs.est.1c00308, PMID: 34101444

[31] Bowes DA Darling A Driver EM Kaya D Maal-Bared R Lee LM. (2023). Structured ethical review for wastewater-based testing. medRxiv [Preprint]. doi: 10.1101/2023.06.12.23291231 PMC10484207 37611169

