# Supplementary material for: A framework for integrating wastewater-based epidemiology and public health
Source: Front Public Health. 2024 Jul 24;12:1418681. doi: 10.3389/fpubh.2024.1418681 (PMC11312382; doi:10.3389/fpubh.2024.1418681)

Supplementary Material for

**A Framework for Integrating Wastewater-based Epidemiology (WBE) and Public Health**

Hanna Brosky^ag^, Sarah M. Prasek^b^, Gabriel K. Innes^a^, Ian L. Pepper^b^, Jasmine Miranda^a^, Paul E. Brierley^a^, Stephanie L. Slinski^a^, Lois Polashenski^b^, Walter Q. Betancourt^b^, Katie Gronbach^a^, Diana Gomez^c^,Reshma Neupane^d^, Jasmine Johnson^d^, Joli Weiss^d^, Hayley Yaglom^e^, David Engelthaler^e^, Crystal Hepp^e^, Katherine Crank^f^, Daniel Gerrity^f^, Jill Stewart^g^, and Bradley W. Schmitz*^a^

^a^Yuma Center of Excellence for Desert Agriculture (YCEDA), University of Arizona, 6425 W. 8^th^ St., Yuma, AZ 85364, USA

^b^Water & Energy Sustainable Technology (WEST) Center, University of Arizona, 2959 W. Calle Agua Nueva, Tucson, AZ 85745, USA

^c^Arizona Department of Health Services, Office of Infectious Disease Services, 150 North 18th Avenue, Suite 140, Phoenix, AZ 85007, USA

^d^Yuma County Public Health Services District, 2200 W 28th St #137, Yuma, AZ 85364, USA

^e^Translational Genomics Research Institute, Pathogen and Microbiome Institute, 3051 W. Shamrell Blvd Ste. 106, Flagstaff, AZ 86005, USA

^f^Applied Research and Development Center, Southern Nevada Water Authority, P.O. Box 99954, Las Vegas, NV 89193, USA.

Current Affiliations:

^g^Environmental Science and Engineering, University of North Carolina, Chapel Hill 135 Dauer Dr, Chapel Hill, NC 27599

Bradley W. Schmitz* Mailing address: 6425 W. 8^th^ Street, Yuma, AZ 85364 Email: bschmitz@arizona.edu

14 Pages

6 Figures

1 Table

Figure S1. Memoranda of Understanding (MOUs)

*Continued on the following page*

 *Continued on the following page*

Figure S2. Steering Committee Communications

*Continued on the following page*

Figure S3. Liability Disclaimer Statement

Liability Disclaimer: Levels of SARS-CoV-2 RNA in wastewater, “Levels of Concern” categorization, results interpretations, and response actions are for risk assessment purposes only. Any response action decisions is solely the responsibility of Yuma County Public Health Services District, County/City administration, municipalities, or any other stakeholders with decision-making capacity (i.e., schools, hospital, etc.). Neither YCEDA nor affiliates shall incur any liability whatsoever for any damage, loss or expenses of any kind suffered or incurred or arising from or incident related to risk assessment and considerations provided.

Figure S4. Public Outreach: Facebook


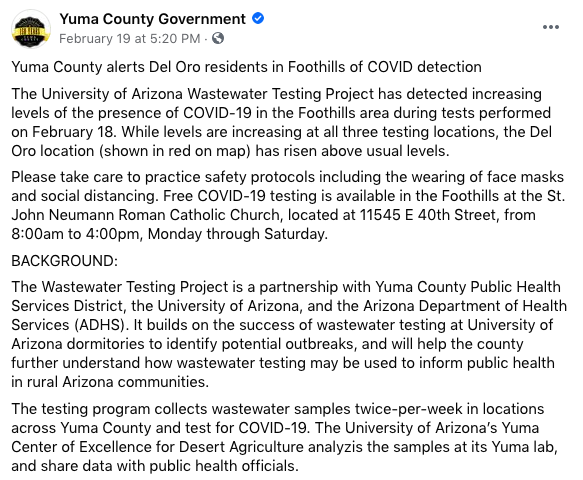


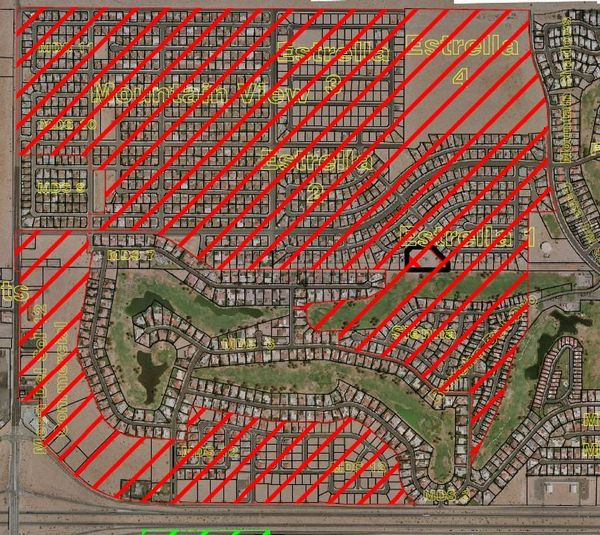


Figure S5. Public Outreach: Media

*Continued on the following page*

*Continued on the following page*

Figure S6. Public Outreach: Press release

*Continued on the following page*

Table S1. Levels of Concern (*Candida auris*)


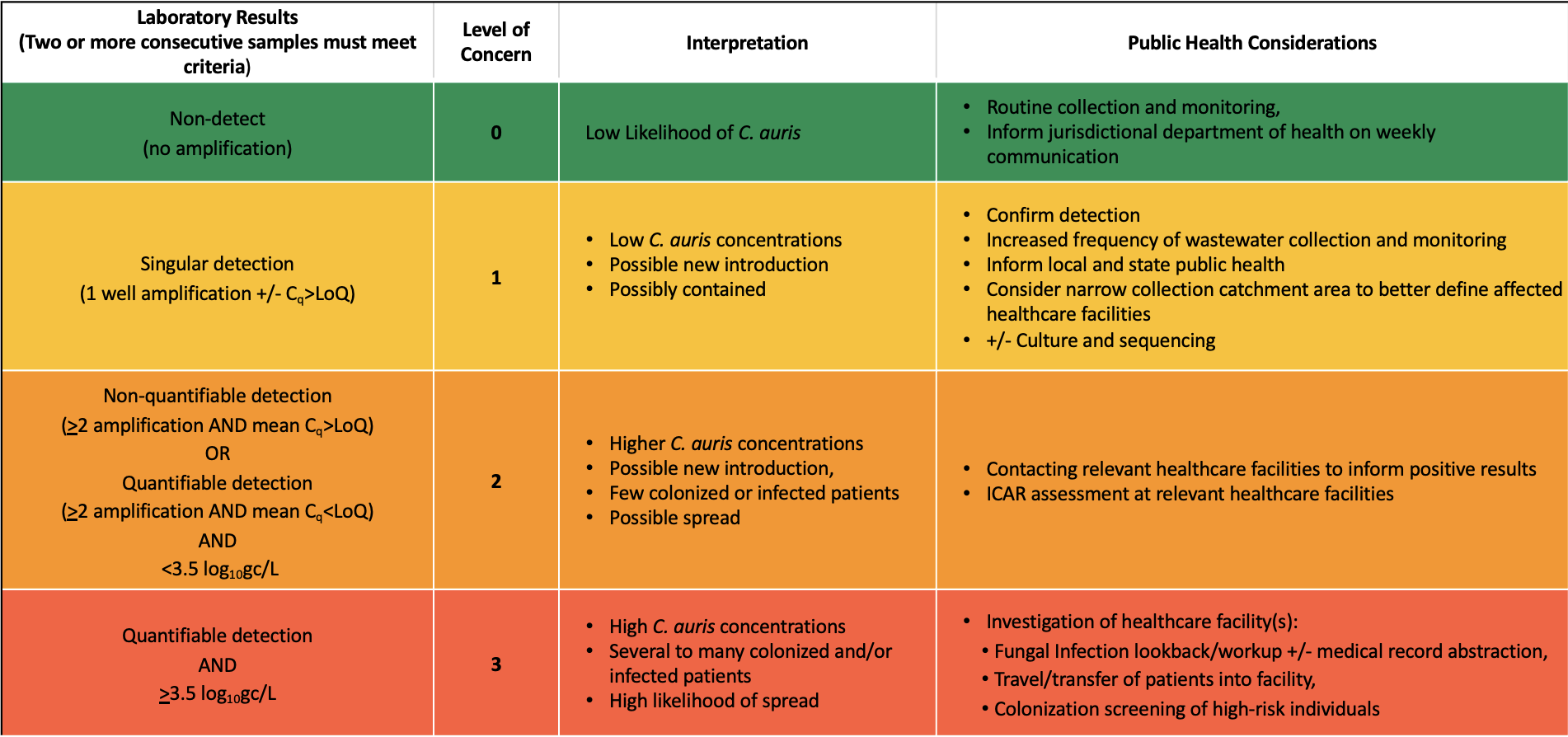

Supplement: Supplementary file 1 [file Data_Sheet_1.docx]
